# Counting drugs to understand the disease: The case of measuring the diabetes epidemic

**DOI:** 10.1186/1478-7954-5-2

**Published:** 2007-02-21

**Authors:** Henrik Støvring, Morten Andersen, Henning Beck-Nielsen, Anders Green, Werner Vach

**Affiliations:** 1 Research Unit of General Practice, University of Southern Denmark, J.B. Winsløwsvej 9A, 5000 Odense C, Denmark; 2 Diabetes Research Center, Odense University Hospital, Kløvervænget 6^4^, 5000 Odense C, Denmark; 3 Department of Epidemiology, University of Southern Denmark, J.B. Winsløwsvej 9B, 5000 Odense C, Denmark; 4 Department of Statistics, University of Southern Denmark, Campusvej 55, 5230 Odense, Denmark

## Abstract

**Background:**

Diabetes prevalence increases globally with severe consequences for afflicted individuals and societies. Data on diabetes incidence and diabetes related mortality on a population level are, however, scarce. As an alternative to dedicated studies it has been suggested to use pharmacoepidemiological databases that are readily available, at least in the Nordic countries.

**Methods:**

For all 470,000 inhabitants in Funen County, Denmark, in the period 1992–2003, data on gender, date of birth, death and migration to and from the county, and any filled prescriptions of an anti-diabetic medication was obtained from the Odense Pharmaco-Epidemiological Database.

**Results:**

Prevalence odds for use of an anti-diabetic medication rose annually 3.5% (95% confidence interval: 3.1%, 3.9%) for females, 4.5% (4.0%, 4.9%) for males. Corresponding incidence rates annually rose 4.8% (3.8%, 5.8%) for females, 4.5% (3.5%, 5.4%) for males. Mortality rates among treated annually declined 2.8% (1.4%, 4.1%) among females, 2.2% (0.9%, 3.5%) among males. The disequilibrium in absolute numbers between incidence and mortality among treated was the main driver for the increasing prevalence, while concurrent trends in incidence and diabetes related mortality only marginally affected prevalence trends. Trend estimates were insensitive to varying the length of the run-in period used for determining treatment status, except when using the naive and methodologically flawed run-in period of variable length.

**Conclusion:**

While pharmacoepidemiological databases provide a useful tool for monitoring pharmacologically treated diabetes, a dedicated diabetes database covering all prevalents and incidents is needed for a more detailed analysis of underlying causes and trends.

## Background

The population epidemiology of diabetes mellitus is of public health interest from several perspectives. Diabetes has severe costs for afflicted patients, as it is a chronic lifelong disease characterized by excess mortality [1] and high comorbidity with cardiovascular disease, nephropathy and eye complications as prominent examples [2]. Diabetes has a high impact on general populations, in particular in Western societies: The prevalence increases with age and reaches a level of about 10% at the age of 60 years and above in most populations [1]. Diabetes prevalence has been found to increase in all age groups during the last 20 years, and WHO has estimated that the number of diabetics in the world will more than double within the next 25 years from about 170 million in 2000 to about 366 million in year 2030 [3]. This increase of about 3.1% per year in prevalence has been claimed to fulfill the criteria for an epidemic, particularly of type 2 diabetes [4,5]. Others have claimed that an increase in prevalence alone cannot be taken as proof of an ongoing epidemic [6]. Also, prevention of diabetes (in particular type II) has attracted much attention with focus on the relation between lifestyle and risk of becoming a diabetic, and several countries have set up large scale prevention programs. From a medical point of view, the secondary prophylaxis is of high interest with focus on improving life quality and survival with diabetes through health initiatives ranging from motivational intervention, monitoring of disease, to new pharmaceutical treatments.

At the heart of these perspectives is the need to obtain valid and current estimates on trends in diabetes incidence, prevalence and mortality. Such estimates are, however, typically costly to obtain on a general population level, and are hence often missing. In this context, it has been suggested that pharmacoepidemiological databases could be used to study the dynamics of the diabetes epidemiology for large, well-defined populations, even when the databases only contain information on filled prescriptions [7]. A prime advantage of using pharmacoepidemiological databases for such epidemiological descriptions is obviously that the data are readily available. But, although other studies have shown that diabetes prevalence may be reliably estimated from total consumption data [8-10], the use of individual claims data for a more detailed analysis of trends in incidence, prevalence and mortality has so far received less attention. Such an approach is not without its challenges, since both date of onset as well as true, current disease status are not recorded in this type of pharmacoepidemiological databases. Misclassification is thus a potential problem, and the type of potential bias introduced by different strategies for determining treatment status at any given point in time must be carefully considered. Indeed, intuitively attractive approaches may introduce strong biases when studying calendar time trends, as discussed below. Although we update and extend previously published results on the epidemiology of diabetes in the Funen population [7], the primary objective of this paper is methodological. First, we investigate the impact of different choices of run-in periods, as this is a crucial methodological issue in many pharmacoepidemiological studies. Secondly, we study the relative contributions of current trends in incidence and mortality to the concurrent, observed rise in diabetes prevalence, ie. we focus on the question: Can prevalence rise while contemporary incidence and mortality rates remain constant?

## Methods

Odense Pharmaco-Epidemiologic Database (OPED) was used as data source. OPED contains information on all redemptions of subsidized and prescribed drugs at community pharmacies in the county of Fyn, Denmark [11] since 1992. It is a copy of the exhaustive electronic register maintained for administering all subsidies to pharmacies as well as prescribing physicians in the county. Information on the drug is entered directly into the electronic register when a prescription is processed and dispensed – which yields very high validity [12] – and in accordance with the Anatomical Therapeutical Chemical (ATC) classification system [13]. All anti-diabetic drugs are characterized by the first three characters being A10, insulin by A10A, oral anti-diabetics by A10B.

OPED contains information on birth date, gender, migration and death for all subjects living within the county in the period January 1, 1992 to December 31, 2003 in accordance with the Danish Central Person Register. All records allow unique tracking of individuals by means of the nationwide Civil Registration Number.

For each individual we determined presence and treatment status with respect to use of anti-diabetic medications on January 1 of each calendar year (the index date). We defined treatment status based on a previous run-in period's recordings of anti-diabetic drug dispensing, ie. if a subject had at least one prescription within the run-in period, the person was considered treated at the index date. Consequently, for each calendar year we excluded all who immigrated into Fyn during the run-in period, since we could not determine treatment status with certainty for immigrants without an observed redemption.

For those present at the index date and with a sufficient run-in period, we computed annual counts of these entities: prevalence (**P**), incidence (**I**), treatment cessation (**C**), deaths among all non-prevalents (**D**_**0**_), deaths among prevalents (**D**_**1**_), and deaths among incidents (**D**_Inc_). An individual was considered prevalent on the index date if a redemption was observed in the associated run-in period. Incidence was defined as the number of subjects having a redemption in the year of interest, but not in the run-in period. Mortality counts were number of deaths in the year of interest stratified on prevalence status at the index date. Among those prevalent at the index date we defined treatment cessation as having been observed for a period which exceeded the length of the run-in period without any new redemptions and which overlapped the index date. In a sub-analysis of the annual transitions between treatment states (treated or not), we further subdivided the number of subjects who had discontinued treatment into two groups: Those who re-initiated treatment within the calendar year, and those who did not.

For incidence and mortality among non-prevalents and prevalents, respectively, we computed rates based on counts and time at risk for the event in question. Time at risk was defined as time from index date to either event or censoring by migration, death, or end of year, whichever was first. Relative mortalities were obtained by comparing mortality rates among prevalents to rates among non-prevalents.

In the sub-analyses of insulin and oral anti-diabetic medications, only redemptions of the specific type were considered; for example, all redemptions of oral anti-diabetic agents were ignored when restricting the analysis to insulin.

Choosing the length of the run-in period is crucial in this study, as in most pharmacoepidemiological studies, since rates of exclusion must be balanced with misclassification. For the fixed run-in periods we did two separate analyses with lengths of one and two years, respectively. The length of one year allows comparison with previously published results [7], but we found it to be inadequate for accommodating a change in the Danish subsidy system taking place in 2000. We thus report on the effects of this change and provide estimates based on a two years run-in period, as these are unaffected by the switch. For illustrative purposes we also present estimates based on increasing the run-in period with calendar time, although these are invalid as they introduce time-dependent misclassification.

As trends in observed incidence of treatment initiation both depends on the true trend in disease incidence as well as trends in detection and tendency to initiate pharmacological treatment, we conducted a sensitivity analysis. Within each year we assume that there is an unknown incidence proportion with an annual trend *ρ*. Further assume that there is a detection probability *π*_*Y*_, ie. of transition from undiagnosed to becoming diagnosed and initiating pharmacological treatment given that one becomes diabetic. We assumed that *π*_1994 _was 60%. Note that results of the sensitivity analysis with respect to trends are virtually insensitive to the actual level of detection, so this choice is not crucial. For changes in this probability we assumed that they were in the range from a decline of 10 percentage points to an increase of 20 percentage points from 1994 to 2003, ie. that detection proportions in 2003 ranged from 50% to 80%. This corresponds to annual changes of -2.0% to 3.2% in detection proportions, ie. a ratio *θ *of 0.980 to 1.032 between subsequent years. The annual trend *ρ *in true incidence can now be related to the observed trend *φ *in incidence of treatment by the following formula

ρ=φθ     (1)
 MathType@MTEF@5@5@+=feaafiart1ev1aaatCvAUfKttLearuWrP9MDH5MBPbIqV92AaeXatLxBI9gBaebbnrfifHhDYfgasaacH8akY=wiFfYdH8Gipec8Eeeu0xXdbba9frFj0=OqFfea0dXdd9vqai=hGuQ8kuc9pgc9s8qqaq=dirpe0xb9q8qiLsFr0=vr0=vr0dc8meaabaqaciaacaGaaeqabaqabeGadaaakeaaiiGacqWFbpGCcqGH9aqpdaWcaaqaaiab=z8aMbqaaiab=H7aXbaacaWLjaGaaCzcamaabmaabaGaeGymaedacaGLOaGaayzkaaaaaa@36AB@

We present graphs showing the relationship between trends in true incidence and the observed trend in treatment incidence when detection proportions vary.

We finally investigated the extent to which the rise in prevalence could be explained by concurrent trends in incidence, mortality among treated and cessation rates, respectively. We did so by comparing the observed annual prevalences to projections based on fixing age-specific rates of incidence, mortality and cessation among treated to their values in 1994. Alternatively, incidence alone was fixed to its level in 1994 to study the isolated impact of a trend in incidence.

### Statistical analysis

Annual incidence, prevalence and mortality rates were determined within four age categories stratified by sex. Annual trend estimates were obtained from regression analysis with year and age categories (cut points at 15, 25, ..., 85) as covariates stratified on gender. Logistic regression was employed for analyzing prevalence, Poisson regression for incidence and mortality rates. In the analyses of prevalence and incidence, correlations among individual's outcomes in subsequent years where allowed for by using robust standard errors [14,15]. For prevalence the trend was estimated as a linear trend on the log-odds-ratio scale, whereas trends in both incidence and mortality were estimated as linear trends on a log-rate-ratio scale. Cessation rates were not analyzed for trends as they are not of primary interest and in any case estimates would be based on small numbers. Estimates of age-adjusted annual trends with 95%-confidence intervals are given as odds-ratios and rate-ratios. All analyses were performed in Stata 8 [16].

## Results

**Figure 1:**
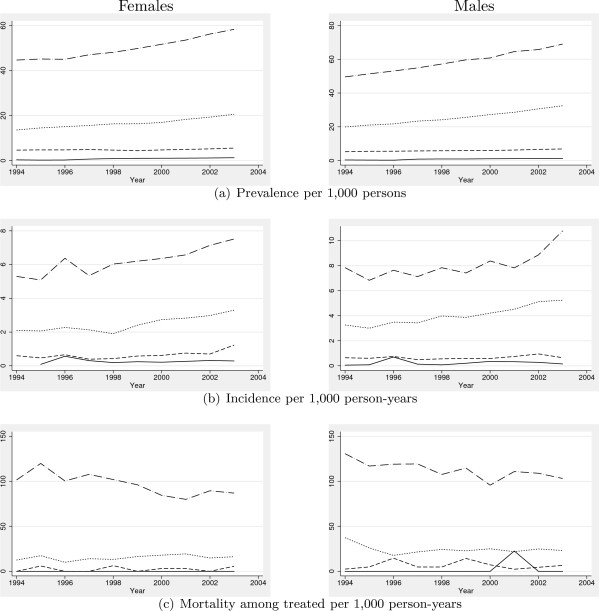
Prevalence, incidence and mortality estimates stratified by gender and age, Fyn County, Denmark, 1994–2003. Line-styles indicate age-categories in years: — 0–14, - - 15–39, ... 40–64, -·- 65+.

Basic characteristics of the studied population are presented in Tables 1 and 2 using a two year run-in period. Population size is almost constant with only a slight increase while the age-composition changes with an increase in the middle-aged group (40–64 years) and among children (<15), and a decline among younger adults (15–39 years). For both sexes, and in all age-groups, prevalence increases, most distinctly among middle-aged, as does incidence, with males aged 15–39 years as the only exception. The total number of deaths among untreated across all age groups declines over the study period, in particular among males above age 65. Mortality among treated increases in absolute numbers, but declines relatively when compared to the concurrent increase in prevalence. A graphical presentation of relative measures is given in Figure 1.

**Table 1:** Characteristics of source population

**Females**	Age																			
	<15					15 – 39					40 – 64					65 >				
				
Year	Size	P	I	*D*_1_	*D*_0_	Size	P	I	*D*_1_	*D*_0_	Size	P	I	*D*_1_	*D*_0_	Size	P	I	*D*_1_	*D*_0_

1994	34,514	10	0	0	8	73,456	339	43	0	39	71,305	967	147	12	318	45,884	2,049	226	197	2,081
1995	34,806	6	3	0	7	72,593	343	33	2	34	72,133	1,046	146	18	313	45,862	2,069	217	234	2,156
1996	35,398	8	20	0	8	71,502	338	46	0	39	73,031	1,099	163	11	325	45,702	2,056	271	196	2,133
1997	35,985	23	11	0	6	70,541	345	27	0	33	73,798	1,149	154	16	311	45,625	2,145	226	219	2,121
1998	36,919	32	7	0	6	69,832	323	29	2	23	74,896	1,221	139	16	314	45,439	2,185	254	211	2,043
1999	37,260	35	9	0	6	69,089	303	39	0	22	75,568	1,234	178	20	323	45,472	2,264	260	206	2,171
2000	37,627	38	8	0	7	68,040	320	41	1	28	76,436	1,293	205	23	310	45,407	2,346	267	190	2,078
2001	38,124	40	10	0	7	66,874	330	49	1	24	77,218	1,412	213	27	308	45,392	2,430	275	187	2,057
2002	38,335	43	12	0	6	66,007	341	45	0	36	77,832	1,500	226	22	276	45,571	2,562	298	219	2,207
2003	38,420	48	11	0	4	65,147	360	78	2	27	78,712	1,620	253	26	307	45,561	2,656	314	221	2,025

**Males**	Age																			

	<15					15 – 39					40 – 64					65 >				
				
Year	Size	P	I	*D*_1_	*D*_0_	Size	P	I	*D*_1_	*D*_0_	Size	P	I	*D*_1_	*D*_0_	Size	P	I	*D*_1_	*D*_0_

1994	36,066	12	2	0	12	77,830	408	49	1	83	71,598	1,424	227	52	478	33,454	1,658	241	203	1,974
1995	36,486	9	3	0	10	76,891	419	45	2	102	72,555	1,525	212	39	451	33,364	1,714	209	189	2,030
1996	37,173	7	26	0	5	75,486	415	55	6	82	73,548	1,597	250	28	448	33,209	1,761	232	197	2,057
1997	37,857	31	5	0	9	74,528	423	36	2	87	74,466	1,739	248	37	455	32,994	1,811	215	203	1,866
1998	38,839	36	3	0	13	73,694	425	40	2	65	75,668	1,826	292	44	471	32,997	1,887	236	192	1,857
1999	39,368	36	8	0	9	72,550	430	42	6	86	76,434	1,956	286	44	455	33,087	1,974	223	213	1,890
2000	39,741	41	14	0	12	71,316	422	41	3	67	77,268	2,103	315	52	463	33,137	2,014	253	184	1,780
2001	39,882	46	13	1	12	70,321	436	51	1	47	77,965	2,227	340	48	444	33,425	2,158	237	226	1,804
2002	40,034	49	11	0	12	69,106	455	64	2	67	78,888	2,414	389	59	477	33,655	2,213	270	228	1,723
2003	40,093	47	6	0	10	68,146	466	43	3	62	79,606	2,583	401	59	469	34,163	2,357	333	230	1,657

Size of population, prevalence, incidence, and mortality with respect to use of anti-diabetic drugs, Fyn County, Denmark, 1994–2003. **Size **is the size of the population on January 1, 1993–2003, where subjects migrating into the capture area in the preceding year are ignored. **P **is the count of prevalent subjects at January 1 of the given year. **I **is the count of incident subjects during the calendar year. **D**_**1 **_is the count of deaths during the year among subjects prevalent at the beginning of the year. **D**_**0 **_are deaths among non-prevalents in the year.

**Table 2:** Characteristics of source population (totals for Table 1)

	Females					Males				
		
Year	Size	P	I	*D*_1_	*D*_0_	Size	P	I	*D*_1_	*D*_0_
1994	225,159	3,365	416	209	2,446	218,948	3,502	519	256	2,547
1995	225,394	3,464	399	254	2,510	219,296	3,667	469	230	2,593
1996	225,633	3,501	500	207	2,505	219,416	3,780	563	231	2,592
1997	225,949	3,662	418	235	2,471	219,845	4,004	504	242	2,417
1998	227,086	3,761	429	229	2,386	221,198	4,174	571	238	2,406
1999	227,389	3,836	486	226	2,522	221,439	4,396	559	263	2,440
2000	227,510	3,997	521	214	2,423	221,462	4,580	623	239	2,322
2001	227,608	4,212	547	215	2,396	221,593	4,867	641	276	2,307
2002	227,745	4,446	581	241	2,525	221,683	5,131	734	289	2,279
2003	227,840	4,684	656	249	2,363	222,008	5,453	783	292	2,198

Total size of population, prevalence, incidence, and mortality with respect to use of anti-diabetic drugs, Fyn County, Denmark, 1994–2003, cf. Table 1. **Size **is the size of the population on January 1, 1993–2003, where subjects migrating into the capture area in the preceding year are ignored. **P **is the count of prevalent subjects at January 1 of the given year. **I **is the count of incident subjects during the calendar year. **D**_**1 **_is the count of deaths during the year among subjects prevalent at the beginning of the year. **D**_**0 **_are deaths among non-prevalents in the year.

Careful inspection of Table 2 reveals that book-keeping with respect to prevalence does not tally in the following sense: The prevalence of a given year does not correspond to the prevalence plus incidence minus deaths among treated of the previous year. What is missing is the count of apparent treatment cessations. In Table 3 the book-keeping is carried out including the annual number of treatment cessations and correcting the number of deaths among the prevalents accordingly. The final column of Table 3 shows that the discrepancy between the predicted prevalence according to this book-keeping principle and the actually recorded prevalence is small. The difference is due to censoring induced by migration, since the cohort is open.

**Table 3:** Book-keeping for size of total prevalent population

Gender	Year	P	I	C_**NI**_	C_**I**_	*D*_1_	*D*_Inc_	Diff
Females	1994	3365	416	107	11	207	18	
	1995	3464	399	123	10	248	12	-3
	1996	3501	500	109	12	206	22	9
	1997	3662	418	94	7	230	23	-24
	1998	3761	429	114	9	225	19	5
	1999	3836	486	91	11	223	14	-15
	2000	3997	521	83	9	209	19	-11
	2001	4212	547	92	8	213	15	-14
	2002	4446	581	96	21	237	20	-8
	2003	4684	656	84	15	244	24	-10

Males	1994	3502	519	94	5	254	23	
	1995	3667	469	109	6	226	18	-6
	1996	3780	563	96	12	228	31	-21
	1997	4004	504	106	7	239	18	-15
	1998	4174	571	105	12	235	26	11
	1999	4396	559	97	11	261	22	-9
	2000	4580	623	80	17	235	26	-17
	2001	4867	641	96	18	274	21	-21
	2002	5131	734	97	25	288	27	-7
	2003	5453	783	111	15	283	39	-27

Bookkeeping for size of total prevalent population with respect to anti-diabetic treatment, Fyn County, Denmark, 1994–2003. **P **is prevalence on Jan 1 of the given year, **I **is number of incident subjects, **C**_**NI **_is number of persons having discontinued treatment without re-initiation in the given year, **C**_**I **_is number of persons having discontinued treatment who re-initiated treatment within the given year, *D*_1 _is number of deaths among treated, and *D*_Inc _is number of incident subjects dying before the end of the year. **Diff **for year *Y *is *P*_*Y *_- (*P*_*Y*-1 _+ *I*_*Y*-1 _- *C*_**NI**, *Y*-1 _- *D*_1,*Y*-1 _- *D*_Inc, *Y*-1_), ie. difference between observed and predicted prevalence. Note, that **C**_**I **_does not enter into the computation of **Diff **since these subjects both leave and re-enter the prevalent pool of subjects in the same year.

Table 4 gives estimates of age- and sex-adjusted trends. Both prevalence and incidence trend estimates are positive and statistically significant, regardless of gender, anti-diabetic drug type, and length of run-in. Mortality declines among treated (except for males with respect to insulin when using a two year run-in period) over the study period, and notably also relative to the non-treated population.

**Table 4:** Trend estimates for incidence, prevalence, and mortality with respect to treatment with anti-diabetic agents

Run-in	Treatment	Gender	Prevalence		Incidence		Mortality among treated		Relative Mortality	
One year	All anti-diabetic	F	1.036	1.032, 1.040	1.045	1.035, 1.055	0.973	0.959, 0.987	0.991	0.986, 0.995
		M	1.044	1.040, 1.048	1.045	1.036, 1.054	0.974	0.961, 0.987	0.977	0.973, 0.982
	Insulin	F	1.036	1.030, 1.043	1.048	1.033, 1.064	0.977	0.955, 1.000	0.990	0.986, 0.994
		M	1.045	1.039, 1.050	1.067	1.053, 1.082	1.000	0.976, 1.023	0.977	0.973, 0.981
	Oral antidiab.	F	1.045	1.039, 1.051	1.052	1.042, 1.059	0.969	0.953, 0.986	0.992	0.987, 0.996
		M	1.052	1.046, 1.058	1.049	1.040, 1.058	0.959	0.943, 0.974	0.979	0.975, 0.984

Two year	All anti-diabetic	F	1.035	1.031, 1.039	1.048	1.038, 1.058	0.972	0.959, 0.986	0.990	0.986, 0.995
		M	1.045	1.040, 1.049	1.045	1.035, 1.054	0.978	0.965, 0.991	0.978	0.974, 0.982
	Insulin	F	1.034	1.028, 1.040	1.049	1.033, 1.065	0.970	0.949, 0.992	0.990	0.986, 0.994
		M	1.045	1.040, 1.051	1.065	1.050, 1.080	1.005	0.983, 1.028	0.977	0.973, 0.982
	Oral antidiab.	F	1.043	1.037, 1.049	1.055	1.045, 1.066	0.970	0.954, 0.987	0.991	0.987, 0.995
		M	1.052	1.046, 1.058	1.050	1.040, 1.060	0.964	0.949, 0.979	0.980	0.976, 0.984

Variable	All anti-diabetic	F	1.046	1.042, 1.050	1.032	1.023, 1.041	0.974	0.962, 0.985	0.991	0.987, 0.995
		M	1.053	1.049, 1.057	1.028	1.020, 1.036	0.973	0.962, 0.984	0.979	0.975, 0.982

Estimated age-adjusted, annual trends with 95%-confidence intervals for different categories of anti-diabetic drugs and with different lengths of run-in periods, Fyn County, Denmark, 1992–2003. For prevalence the trend estimate is given as an odds-ratio based on logistic regression, whereas the remaining estimates are rate-ratios based on Poisson regression. The start of the study period depends on the length of run-in period used.

### Methodological considerations I: Length of run-in

Table 5 shows the annual count of patients defined as having discontinued treatment based on a gap of either one year or two years between last redemption in the run-in period and first redemption in the following year. Using a gap of one year leads to a peak in 2001 reflecting a change in reimbursement policy in 2000, so apparently some have stockpiled in early 2000 followed by a longer period without redemptions. This effect is smoothed out by using a gap of two years, i.e. the two year run-in period defines treatment status without obvious time-dependent misclassification.

As seen from Table 4 there is, however, virtually no difference in trend estimates of prevalence, incidence and mortality, whether we choose a one or two year year long run-in period. Only exception is for incidence, and this is a result of a high incidence in 1993. With a two year run-in period, we are forced to omit this year when estimating trends, and hence the trend is attenuated.

**Table 5:** Discontinuation of anti-diabetic treatment

	Females				Males			
		
	One year gap		Two years gap		One year gap		Two years gap	
Year	*n*	%	*n*	%	*n*	%	*n*	%
1993	198	6.6			174	5.6		
1994	209	6.7	118	3.5	185	5.7	99	2.8
1995	199	6.2	133	3.8	188	5.5	115	3.1
1996	172	5.3	121	3.5	203	5.8	108	2.9
1997	188	5.5	101	2.8	196	5.2	113	2.8
1998	163	4.6	123	3.3	182	4.6	117	2.8
1999	135	3.7	102	2.7	166	4.0	108	2.5
2000	150	4.0	92	2.3	194	4.5	97	2.1
2001	282	7.1	100	2.4	375	8.2	114	2.3
2002	176	4.2	117	2.6	233	4.8	122	2.4
2003	189	4.2	99	2.1	250	4.9	126	2.3

Number of patients discontinuing anti-diabetic treatment, Fyn County, Denmark, 1994–2003. Discontinuation is defined as subjects with more than one or two years, respectively, without redemptions after a previous redemption. Percentage figures indicate relative proportion of the prevalent population who discontinues treatment.

If, however, we were to naively use all available information prior to a given index date to determine treatment status, trend estimates of prevalence become markedly increased, and trend estimates for incidence decreased. The relative biases of this naive approach range from 20%–40% for these parameters. Trends in mortality are unaffected by using a variable length run-in period.

### Methodological considerations II: Trends in detection proportions

**Figure 2:**
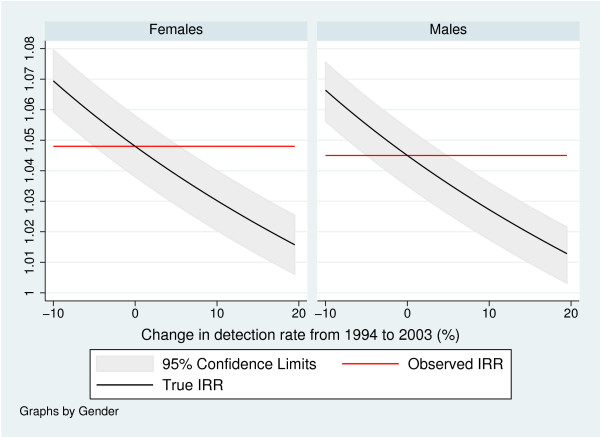
Observed and true trends in incidence with respect to hypothesized changes in detection proportions from 1994 to 2003. Confidence limits for the true trends are computed from the confidence limits of the observed trend by application of Formula 1.

In Figure 2 we present the results of our sensitivity analysis. As observed trends in incidence we used the estimated trends of 1.048 (1.03.8, 1.058) for females, and 1.045 (1.035, 1.054) for males. Even for unrealistic large changes in detection proportions the trend in true incidence does not vanish.

### Methodological considerations III: Anatomy of an "epidemic"

**Figure 3:**
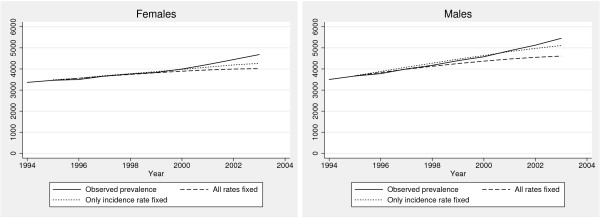
Observed and predicted prevalences with respect to use of anti-diabetic drugs, Fyn County, Denmark, 1994–2003. Fixation of rates is with respect to their 1994-level.

Above we observed an increase in prevalence and incidence accompanied by a decrease in mortality among treated. A common interpretation would be to explain the trend in prevalence with the concurrent trends in incidence and mortality. This would however be an oversimplification if prevalence could rise while incidence and mortality rates remained constant. To examine whether this actually occurred during the study period, we compared the observed number of prevalents over the study period to the number of prevalents that would be predicted if incidence, mortality among treated, and cessation rates all had remained constant at their level in 1994. Alternatively, we only fixed incidence rate at its 1994-level, while allowing mortality among treated and cessation rates to vary as observed. The resulting projections are shown in Figure 3.

For the period 1995–1999, agreement is very good between observed and projected prevalence among females, while poorer among males. For both genders the discrepancy increases from year 2000. Keeping only incidence fixed improves agreement between observed and projected prevalence substantially, in particular among men, ie. for men the combined decline in mortality and cessation explain more of the observed rise in prevalence than for women. The bulk of the rise in prevalence is thus not due to contemporary changes in incidence or mortality rates, but to incident subjects outnumbering deaths among treated and treatment cessations.

## Discussion

Throughout the study period, prevalence of use of anti-diabetic agents increased both in absolute and relative terms, as well as within and across age strata. The corresponding incidence also increased, while mortality among treated declined, also relative to mortality among untreated. This agrees with previous results [7], except with respect to incidence.

The data used are generally of high validity, as they result from automated registration of economic transaction data; near complete coverage, except for medications filled at a pharmacy outside the subjects residential county, which for all drugs was found to not exceed 0.5% [12]; the information on migration and vital statistics is identical to the relevant subset of the registration at Statistics Denmark.

The major limitation of the present study is its reliance on drug redemptions. Obtaining a drug is not equivalent to using it, and when used there is no observation of end of use. Consequently, we investigated the impact of different treatment classification rules and found that trend estimates for prevalence, incidence, and mortality were largely unaffected, except for the flawed principle of using a run-in period of varying length.

Further, our estimates for prevalence, incidence, and mortality by definition only concern subjects with treated diabetes, i.e. our results do not apply to undiagnosed diabetics, nor to diabetics treated exclusively with diet. These subjects are misclassified in the sense that they are considered part of the general, non-diabetic population in the present study. As all Danish citizens are covered by a government funded health care plan, the annual cost of medications for chronic diseases to be paid by a single individual cannot exceed approximately DKK 3,600 (nearly USD 600) per year. In contrast to many other countries one may therefore expect a rather limited dependence of misclassification rates on social class and, in particular, financial capacity. Other heterogeneities in misclassification rates due to life style, attitudes to health care, etc., may also exist, but like those due to financial capacity and social class, we have little reason to assume that they have changed substantially over the last decade and could imply artificial time trends in incidence or prevalence. Especially, we did not identify the age trend in cessation rates reported by Glynn *et al *in two studies [17,18]. Only for the youngest age-groups were cessation rates slightly elevated, and this may indicate that misclassification may not so much depend on cost as on need and perceived necessity of treatment.

The prevalence level of pharmacologically treated diabetes found here is in line with other contemporary studies in Denmark [19,20]. For the year 1996, Kristensen *et al *[21] found that 71% of all patients diagnosed with type II diabetes in Vejle County, Denmark, were treated pharmacologically. Further, Drivsholm *et al *[19] estimated from a one year run-in period the number of pharmacologically treated diabetes patients in Denmark on Dec 31, 1999, to 98,358, constituting about 75% of the population of all identified diabetics, estimated to about 130,000 on the same date. Interestingly, Drivsholm *et al *also found that their primary data sources on hospital discharge diagnosis, measurement of blood glucose, and diabetic foot care, were only able to identify approximately 80%–90% of patients treated pharmacologically for diabetes, indicating the lack of a single data source with complete coverage. In short, it appears reasonable to consider the proportion of pharmacologically treated diabetes patients constant across the study period at about 75%, and to expect the estimated trends in prevalence of pharmacologically treated diabetes to be valid for the entire diabetes population. Regardless, we suggest our results to be of genuine public health interest in themselves, as they pertain to that group of diabetics which must generally be considered most severely afflicted, and thus requiring most care.

A third consequence of relying on a pharmacoepidemiological database is the inability to control for date of diagnosis in the analysis. In particular, in the analysis of mortality among treated we could not account for duration of disease, and part of the explanation for the apparently decreasing mortality could be earlier treatment of patients with less severe diabetes.

The trend in incidence identified in this study was comparable to the one found for the period 1993–1999, reported in [7], although slightly attenuated. The attenuation occurred both because the year 1993 was excluded in the present study using a two year run-in period, and because incidence began a steeper increase in 2000. Combined with the enlarged sample size due to a longer study period, this made the trend statistically significant. The increase in incidence observed after year 2000 could hallmark the onset of a true diabetes epidemic, possibly reflecting an impact from a sustained obesity epidemic on onset of diabetes or other changes in lifestyle – although postulating a general obesity epidemic in Denmark would also appear to be an over-simplification according to a recent study showing the existence of large heterogeneity in obesity trends [22]. That an epidemic of diabetes could be looming was supported by the finding that fixing incidence at its level in 1994 could predict prevalence with good precision until 2000, at which point the observed prevalence started to outrun projected prevalence. It may, however, be speculated that new diagnostic criteria, which eased detection of new cases and were implemented in 1999 in Denmark, could have contributed to the observed increase in incidence. For trends in incidence, our somewhat simplistic sensitivity analysis did however show that even unrealistically large changes in detection proportions could not remove the observed trend in incidence entirely. One must however interpret these results cautiously for two reasons. First the detection proportions are by definition a simplified representation of the problem, as they ignore the duration between true onset of disease and initiation of pharmacological treatment. We are however not aware of any studies on this duration, let alone trends in durations, which could be used to further qualify our analysis, as existing studies only report prevalence of undiagnosed diabetes [23]. Secondly, the detection proportion covers both diagnosis and treatment initiation. It might well be speculated that a trend towards earlier diagnosis could be offset by a smaller need for pharmacological treatment among those with earlier diagnosis. For this reason we believe that the studied range of changes in detection proportions is probably too wide to only contain realistic values. Note, that our considerations about the relation of observed incidence, prevalence and mortality are independent of any assumptions about the causal reason for a shift in incidence, be it a real increase in subjects developing diabetes, a change in diagnostic procedures, criteria or habits, or a change in treatment practices.

Whatever the explanation, the trend in incidence should not be considered the primary reason for the concurrent increase in prevalence. Instead, the primary explanation is the annual surplus of subjects initiating treatment for diabetes compared to those leaving treatment which must be considered a legacy of past diabetes incidence and treatment. The surplus may be expected to increase further, if current trends in incidence and mortality persist, and projections by WHO and others may thus severely underestimate the true magnitude of diabetes prevalence ten to twenty years from now, built as they are on stationary age-specific incidence and mortality estimates [3].

In conclusion, we suggest that pharmacoepidemiologic databases can provide useful information on current trends in diabetes epidemiology if analyzed properly, and at a much lower cost than ordinary studies. The major drawback is the lack of insight in the underlying reasons for any observed trends – an insight which can only be obtained on a regular basis if a population-wide case database collecting information on all incident and prevalent diabetes cases is established.

## Competing interests

The author(s) declare that they have no competing interests.

## Authors' contributions

Støvring had the original idea for the study, carried out all analyses and drafted the original and revised manuscripts. Andersen provided access to the data. The planning of analyses and interpretation of the data was the product of discussions involving all authors, all of whom also commented on drafts of the paper. All authors have seen and approved the final version.
